# Comparison between MRI FLAIR vascular hyperintensity-DWI mismatch and perfusion based triage for thrombectomy in the late time window

**DOI:** 10.3389/fneur.2024.1400524

**Published:** 2024-07-25

**Authors:** Leilei Luo, Guanen Zhou, Fanlei Meng, Shuling Liu, Sifei Wang, Yuchao Dou, Da Lu, Ming Wei

**Affiliations:** ^1^Department of Neurology, Tianjin Huanhu Hospital, Tianjin, China; ^2^Department of Neurosurgery, The Second Hospital of Tianjin Medical University, Tianjin, China; ^3^Department of Neurosurgery, Tianjin Huanhu Hospital, Tianjin, China; ^4^Department of Academy of Medical Engineering and Translational Medicine, Tianjin University, Tianjin, China

**Keywords:** FVH-DWI mismatch, perfusion, endovascular thrombectomy, acute ischemic stroke, triage

## Abstract

**Background:**

The clinical impact of patient selection using FLAIR vascular hyperintensity (FVH)–diffusion-weighted imaging (DWI) mismatch for endovascular thrombectomy (EVT) in patients who have been symptomatic for over 6 h remains unclear. Herein, a retrospective study was conducted to compare the inter-rater reliability and clinical outcomes of patients selected for thrombectomy based on FVH-DWI mismatch with perfusion.

**Methods:**

Patients with anterior-circulation large-vessel occlusion selected simultaneously with MRI and perfusion imaging in the late time window from a single-center retrospective study were categorized into EVT-applicable (FVH-DWI mismatch on MRI or perfusion imaging meeting the DEFUSE3 standards) and EVT-inapplicable groups based on MRI and perfusion imaging. The primary outcome was the 90-day functional independence rate. Safety outcomes encompassed symptomatic intracranial hemorrhage and mortality in 90 days. We assessed the consistency of the two profiles and compared the differences in functional independence rates of EVT patients among the EVT-applicable groups determined by MRI and perfusion.

**Results:**

A total of 130 patients were enrolled, of which 114 were classified into the EVT-applicable group after triaging using MRI images. In this group, 96 patients underwent EVT, with 53 of them (55.2%) achieving functional independence. A total of 110 patients were divided into EVT-applicable group based on perfusion, among which 92 underwent EVT, with 49 of them (53.2%) achieving functional independence. The consistency of identifying EVT indication was moderate between two groups (κ = 0.42, 95% CI, 0.17–0.67). The functional independence rate was comparable between patients in the two EVT-applicable groups based on the two methods (55.2% vs. 53.2%, *p* = 0.789).

**Conclusion:**

MRI triaging based on FVH-DWI mismatch showed moderate inter-rater reliability compared with perfusion-based triage and comparable efficacy in predicting clinical outcomes after EVT.

## 1 Introduction

Numerous randomized controlled trials (RCTs) have verified the effectiveness and safety of endovascular thrombectomy (EVT) in patients with acute ischemic stroke (AIS) caused by large-vessel occlusion (LVO) of the anterior circulation (1, 2). In the DEFUSE 3 and DAWN trials, computed tomography perfusion (CTP) or magnetic resonance diffusion and/or perfusion was performed, and the infarct core and ischemic penumbra were calculated using the Rapid Processing of Perfusion and Diffusion (RAPID; iSchema View, Menlo Park, CA, United States) software (1, 2) in the late time window (6–24 h). However, access to perfusion in emergencies is expensive and not readily available in many stroke centers globally (3). Additionally, the selection range of perfusion screening is narrow, resulting in the omission of patients who could benefit from thrombectomy surgery (4). Therefore, a simpler and more feasible image evaluation method is needed in clinical practice (5).

Assessment based on collateral circulation is currently a popular method, and better treatment benefits for EVT patients with good collateral circulation have been demonstrated in post-hoc analyses of the MR CLEAN, SWIFT, and DAWN trials (6–8). The subsequent MR CLEAN LATE study also confirmed the efficacy of CTA assessment of collateral circulation for patients with anterior circulation large vessel occlusion who presented 6–24 h from onset, but more symptomatic intracranial hemorrhage (4). The FVH sign in the fluid-attenuated inversion recovery (FLAIR) sequence is a simpler method with which to evaluate collateral circulation without the need for contrast agents and intelligent software processing. Described as a focal, undulating, or linear hyperintensity, this anomaly typically manifests in the Sylvian fissure and is linked to large-vessel occlusion or stenosis (9). FVH most likely indicates slow arterial blood flow, with possible mechanisms including slow retrograde flow in the leptomeningeal collaterals or antegrade flow in the setting of hemodynamic compromise. An FVH-DWI mismatch was considered when the FVH exceeded the perimeter of the DWI cortical lesion. Some studies have confirmed that FVH-DWI mismatch shows excellent sensitivity and specificity in predicting PWI-DWI mismatch, and is thus expected to become a new target for EVT selection (10).

We sought to evaluate the impact of MR imaging selection modality on the clinical outcomes of EVT, and the interrater reliability of MRI and perfusion imaging selection modalities in a retrospective study.

## 2 Materials and methods

### 2.1 Patient population

TRACK-LVO (Triage of Patients With Acute Ischemic Stroke Due to Large Vessel Occlusions: An Imaging-based Patient Registry Study; NCT 05659160) registry is an ongoing, prospective, multicentre trial registry of consecutive patients with large vessel occlusion undergoing endovascular thrombectomy or medication only. In this study, a retrospective analysis was conducted of 130 patients with acute ischemic stroke with anterior-circulation large-vessel occlusion treated between January 2019 and October 2022 in Tianjin Huanhu hospital based on TRACK-LVO. Only those patients meeting the specific criteria were included, namely: (1) aged 18 years or older; (2) presenting with symptoms consistent with acute ischemic stroke within 6–24 h of onset; (3) with a CT Alberta Stroke Program Early Computed Tomography Score (ASPECTS) of 6 or higher, a National Institutes of Health Stroke Scale (NIHSS) of 6 or higher, and a modified Rankin scale (mRS) of 0–2 before the stroke; (4) with a definite diagnosis of occlusion in the internal carotid artery (ICA) and/or the M1 segment of the middle cerebral artery (MCA-M1) and M2 proximal segment; (5) having undergone complete MRI, CTP, or MRI perfusion at the same time. The exclusion criteria included incomplete three-month follow-ups and imaging data that could not be analyzed.

### 2.2 Methods

#### 2.2.1 Data assessment and surgical indication

**Figure 1:**
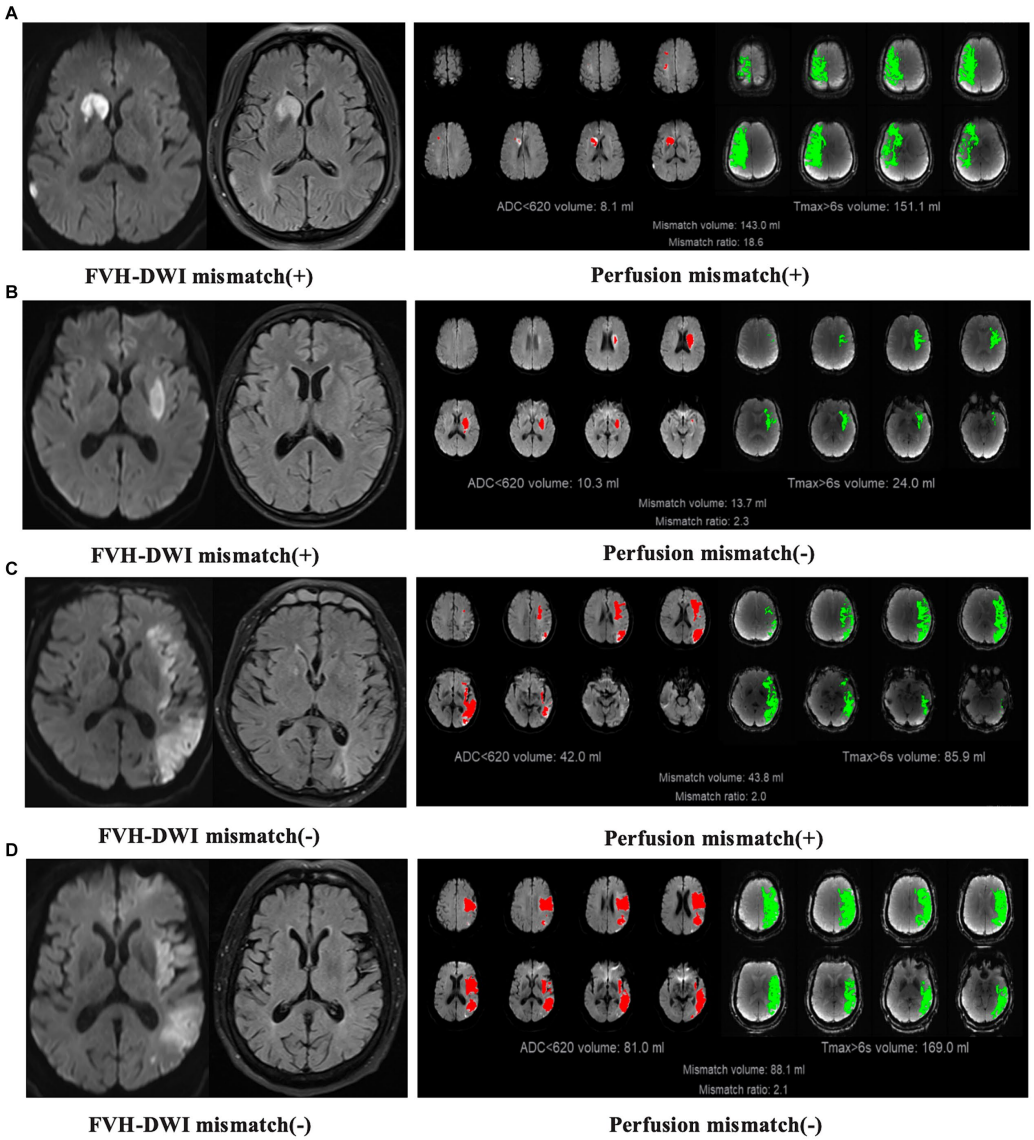
Examples illustrating the concordance and discordance between FVH-DWI mismatch and perfusion imaging. **(A)** FVH-DWI mismatch(+) and perfusion(+). **(B)** FVH-DWI mismatch(+) and perfusion(−). **(C)** FVH-DWI mismatch(−) and perfusion(+). **(D)** FVH-DWI mismatch(−) and perfusion(−). FVH-DWI mismatch(+) is defined when FVH extends beyond the boundaries of cortical DWI lesions on axial FLAIR and DWI images (when ≥1 FVH is of equal intensity on the DWI). Perfusion(+) is defined when there is low regional cerebral blood flow (<30%) < 70 mL with mismatch ratio ≥ 1.8 and mismatch volume ≥ 15 mL.

All images were interpreted by independent, blinded, neuroimaging core laboratory personnel. Three raters independently evaluated the MRI and perfusion images, reaching an agreement on the FVH-DWI mismatch. All patients who met the inclusion criteria were evaluated for EVT indication by MRI and perfusion imaging, respectively. When screened by MRI, patients with FVH-DWI mismatches were considered to have EVT indication, otherwise there was no EVT indication. The FVH sign was defined as focal, tubular, or serpentine hyperintensity in the lateral fissure, sulcus, or near the surface of the brain on the FLAIR sequence (9). A FVH-DWI mismatch was identified where FVH extended outside the borders of cortical DWI lesions on the axial FLAIR and DWI images (when ≥1 FVH was of equal density on DWI; Figure 1). When perfusion screening was performed, patients with perfusion mismatch (When the regional cerebral blood flow (<30%) was <70 mL with a mismatch ratio ≥ 1.8 and a mismatch volume ≥ 15 mL) were considered to have EVT indication, otherwise there was no EVT indication. All perfusion data were analyzed using F-STROKE software (version 1.0.23; NeuroBlem Ltd. Co.; Figure 1).

#### 2.2.2 Endovascular therapy

Experienced neurologists decided to proceed with EVT according to the clinical guidelines and hospital operating standards, combined with the NIHSS score, ASPECTS score, FVH-DWI mismatch, and perfusion imaging (11). All patients underwent standard endovascular thrombectomy and medical management. EVT was performed by physicians with more than 5 years of experience. At present, Solitaire TMAB/FR (Medtronic) combined with a suction catheter is the preferred method for thrombectomy under conscious sedation (12). For patients with atherosclerotic stenosis after thrombectomy, balloon dilatation can be employed independently or in conjunction with stent placement, if necessary. The use of tirofiban, alteplase, or other medications was determined at the discretion of the physician based on available clinical, imaging, and procedural information. The usage and dosage of rtPA and tirofiban were in accordance with guidelines and expert consensus recommendations. Before EVT, anteroposterior and lateral imaging was performed in a standard manner to assess vascular occlusion. After EVT, standard anteroposterior and lateral imaging were performed to evaluate reperfusion. In general, the number of passes did not exceed five.

#### 2.2.3 Outcome measures

The primary outcome was the rate of functional independence at 90 days (mRS score ≤ 2; mRS scores range from 0 to 6, with higher scores indicating more severe disability). Safety outcomes included: (1) symptomatic intracranial hemorrhage (sICH), defined according to the Heidelberg bleeding classification (13) (an increase in the NIHSS score by 4 points or an increase in the score within a specific NIHSS subcategory by 2 points in conjunction with any intracranial hemorrhage found on imaging); (2) 90-day mortality. Professionals who were unaware of the imaging grouping conducted structured telephone interviews using standardized forms during follow-up.

#### 2.2.4 Statistical analysis

Statistical analysis was performed using SAS software (version 9.4). Categorical variables were presented as frequencies and proportion (%), while the mean ± SD or median ± interquartile range was applied to describe continuous variables. The concordance and discordance between FVH-DWI mismatch and the perfusion imaging profiles were assessed using Cohen κ. The functional independence rates were compared between groups using logistic regression models. Univariate comparisons were made for dichotomized outcomes (mRS 0–2, mTICI, and sICH). The *p*-values that were reported were two-sided, with values less than 0.05 being deemed statistically significant.

## 3 Results

**Figure 2:**
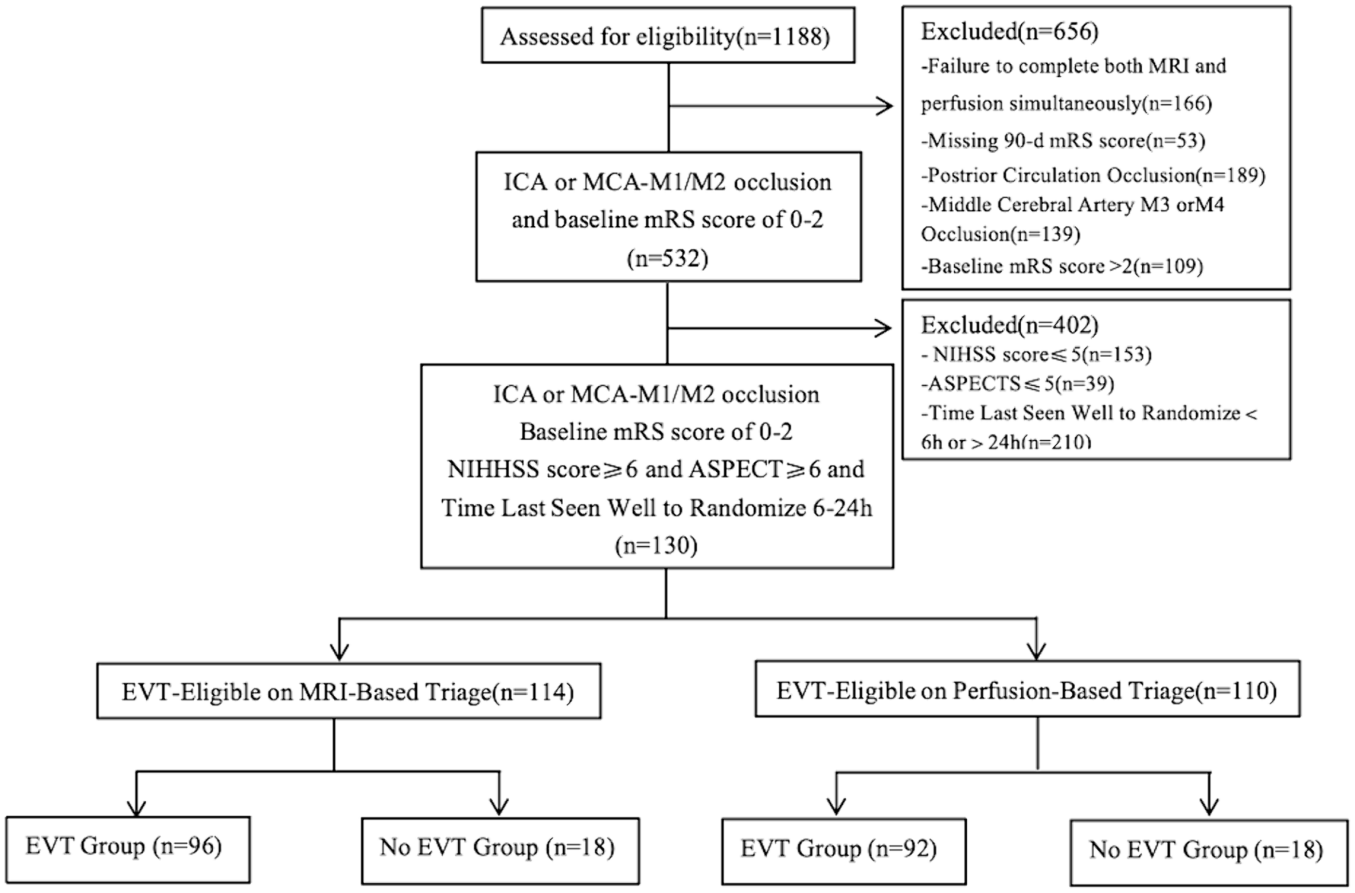
Study flow diagram. ICA, internal carotid artery; MCA, middle cerebral artery; mRS, modified Rankin Scale; NIHSS, National Institutes of Health Stroke Scale; ASPECT, Alberta Stroke Program Early Computed Tomography Score.

A total of 1,188 cases from the TRACK-LVO cohort were assessed for eligibility, of which 532 had ICA or MCA-M1/M2 occlusion and a baseline mRS score of 0–2. In the end, 130 cases had a time from stroke onset of 6–24 h, with a NIHSS score of ≥6 points and an ASPECT score of ≥6 points (Figure 2). The median age of the patients was 63 years (interquartile range, 52–68 years). Among the patients, 54.6% were male. The median NIHSS score was 11 (interquartile range, 8–15), while the median CT ASPECTS was 8 (interquartile range, 7–9). The infarction core volume was 9 mL (interquartile range, 0–24.95) and the ischemic penumbra volume was 118 mL (interquartile range, 80–178; Table 1).

**Table 1:** Baseline characteristics and outcomes of total patients and EVT-eligible groups on MRI- and perfusion-based triages.

Characteristic	Total (*N* = 130)	EVT-applicable group on MRI-based triage (*N* = 114)			EVT-applicable group on perfusion-based triage (*N* = 110)		
		EVT (*n* = 96)	No EVT (*n* = 18)	*P*	EVT (*n* = 92)	No EVT (*n* = 18)	*P*
Age Mean (IQR)	63 (52, 68)	60.5 (51, 68)	64 (56.5, 68.75)	0.16	63 (52.75, 68.25)	64 (55.25, 68.75)	0.56
Male (%)	71 (54.6%)	54 (56.2%)	9 (50%)	0.797	51 (55.4%)	10 (55.6%)	1
CT ASPECTS (IQR)	8 (7, 9)	8 (7, 9)	8 (7, 9)	0.178	8 (7, 9)	8 (7.25, 9)	0.805
DWI ASPECTS (IQR)	7 (6, 8)	6 (5, 8)	7 (7, 8)	0.014	6.5 (5, 8)	8 (7, 8)	0.003
FVH ASPECTS (IQR)	3 (2, 4)	3 (2, 4)	4 (2.25, 4.75)	0.089	3 (2, 4)	4 (2.25, 5)	0.014
NIHSS on admission (IQR)	12 (8, 15)	11 (8, 15)	12 (9.5, 14)	0.512	11 (8, 15)	12 (9.5, 14)	0.734
NIHSS at discharge Median (IQR)	9 (5, 12)	8 (5, 11)	11 (8.25, 13.5)	0.012	8 (5, 11)	10.5 (8.25, 13.5)	0.018
Infarction core volume (IQR)	9 (0, 24.95)	9 (0, 24.72)	13.3 (0, 22.5)	0.865	7.5 (0, 21.25)	13.3 (0, 22.5)	0.441
Ischemic penumbra volume (IQR)	118 (80, 178)	115 (77.4, 168.5)	134 (100.25, 274.70)	0.085	118.3 (84.75, 168)	134 (100.25, 249.38)	0.141
From stroke onset to puncture (IQR)	NA	763 (645.50, 1,020)	NA	NA	763 (623.25, 1012.5)	NA	NA
From puncture to recanalization (IQR)	NA	80 (62.75, 104)	NA	NA	80.5 (63.75, 109)	NA	NA
ASITN/SIR (IQR)	NA	2 (1, 3)	NA	NA	2 (0, 3)	NA	NA
Functional independence rate at 90 days (%)	59 (45.5%)	53 (55.2%)	4 (22.2%)	0.019	49 (53.3%)	4 (22.2%)	0.02
Mortality at 90 days (%)	9 (6.9)	5 (5.2%)	1 (5.6%)	1	7 (7.6%)	1 (5.6%)	1

IQR, interquartile range; CT, computed tomography; DWI, Diffusion-weighted image; FVH, FLAIR Vascular Hyperintensity; ASPECT, Alberta Stroke Program Early Computed Tomography Score; NIHSS, National Institutes of Health Stroke Scale; ASITN/SIR, American Society of Interventional and Therapeutic Neuroradiology/Society of Interventional Radiology.

Of the 130 patients screened using the MRI findings, 114 (87.7%) were eligible for EVT, among which EVT was performed in 96 (84.2%). Based on perfusion screening, 110 patients (84.6%) were considered eligible for EVT. Subsequently, EVT was performed in 92 of these patients (83.6%). A total of 103 patients were applicable for EVT both by MRI and perfusion screening. Eleven patients were applicable for EVT based on MRI but not based on perfusion, while seven patients were applicable for EVT based on perfusion but not based on MRI. Nine patients were EVT-inapplicable both based on MRI and perfusion. The consistency of identifying EVT indication was moderate between the two methods (κ = 0.42, 95% CI, 0.17–0.67).

**Figure 3:**
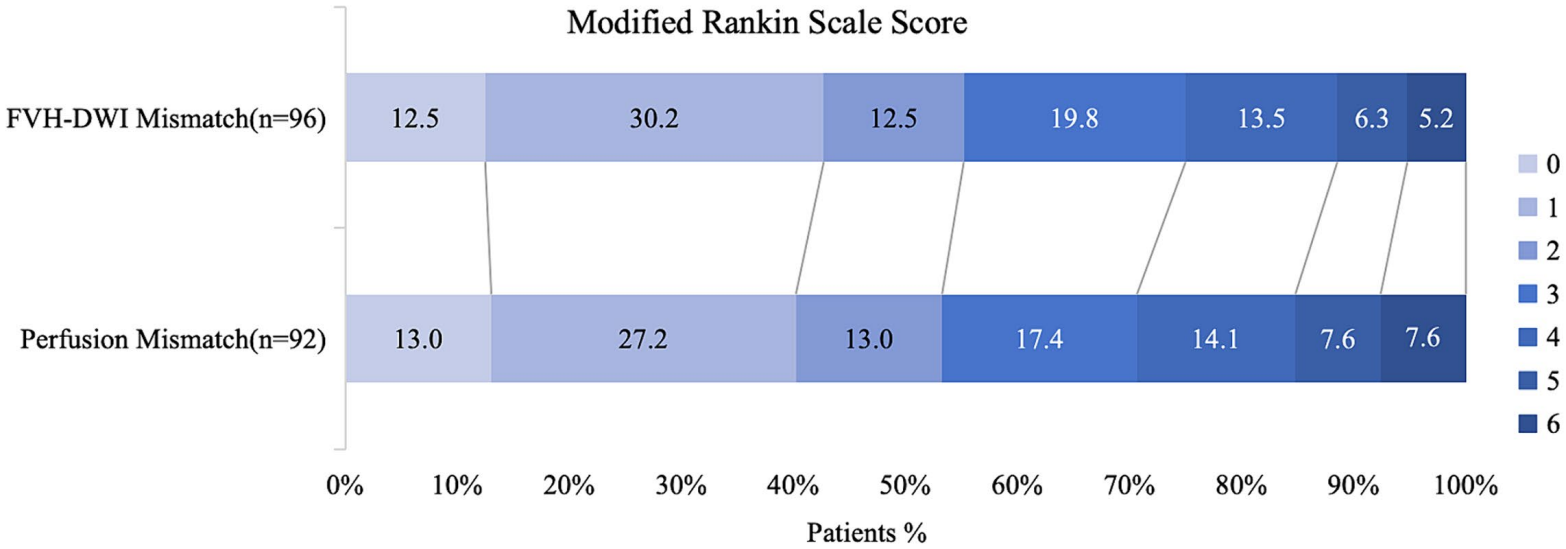
Distribution of functional outcomes at 90 days in patients selected by the presence of FVH-DWI mismatch compared to perfusion mismatch. Functional independence rates were comparable among EVT patients in two EVT-applicable groups which screening through MRI and perfusion, respectively (55.2% vs. 53.2%, *p* = 0.789).

Among the EVT-applicable groups based on the MRI findings, the functional independence rate of patients with EVT was significantly higher than that of patients without EVT (55.2% vs. 22.2%, *p* = 0.019; Table 1). Among the EVT-applicable groups based on perfusion, patients who underwent EVT had a significantly higher rate of functional independence than those who did not (53.2% vs. 22.2%, *p* = 0.02; Table 1). The functional independence rates were comparable among EVT patients in two EVT-applicable groups screened through MRI and perfusion, respectively (55.2% vs. 53.2%, *p* = 0.789; Table 2; Figure 3). No significant differences were observed in the safety outcomes for sICH (5.2% vs. 4.3%, *p* = 0.783; Table 2) and mortality (5.2% vs. 7.6%, *p* = 0.503; Table 2).

**Table 2:** Clinical and imaging outcomes in thrombectomy-treated patients by imaging profiles.

Outcome		FVH-DWI Mismatch (+)	Perfusion Mismatch(+)	FVH-DWI Mismatch(+)/Perfusion Mismatch(+)	FVH-DWI Mismatch(+)/Perfusion Mismatch(−)	FVH-DWI Mismatch(−) /Perfusion Mismatch(+)	FVH-DWI Mismatch(−) /Perfusion Mismatch(−)	*p*-value (B vs. C)
		(*N* = 96)	(*N* = 92)	(*N* = 85) (A)	(*N* = 11) (B)	(*N* = 7) (C)	(*N* = 3) (D)	
90-day modified Rankin Scale score	0	12 (12.5%)	12 (13.0%)	12 (14.1%)	0 (0.0%)	0 (0.0%)	0 (0.0%)	0.052
	1	29 (30.2%)	25 (27.2%)	23 (27.1%)	6 (54.5%)	2 (28.6%)	0 (0.0%)	
	2	12 (12.5%)	12 (13.0%)	12 (14.1%)	0 (0.0%)	0 (0.0%)	0 (0.0%)	
	3	19 (19.8%)	16 (17.4%)	16 (18.8%)	3 (27.3%)	0 (0.0%)	3 (100%)	
	4	13 (13.5%)	13 (14.1%)	12 (14.1%)	1 (9.1%)	1 (14.3%)	0 (0.0%)	
	5	6 (6.3%)	7 (7.6%)	5 (5.9%)	1 (9.1%)	2 (28.6%)	0 (0.0%)	
	6	5 (5.2%)	7 (7.6%)	5 (5.9%)	0 (0.0%)	2 (28.6%)	0 (0.0%)	
mRS score ≤ 2		53 (55.2%)	49 (53.2%)	47 (55.3%)	6 (54.5%)	2 (28.6%)	0 (0.0%)	0.287
sICH		5 (5.2%)	4 (4.3%)	4 (4.7%)	1 (9.1%)	0 (0.0%)	1 (33.3%)	0.999
mTICI 2b − 3		87 (90.6%)	84 (91.3%)	77 (90.6%)	10 (90.9%)	7 (100.0%)	3 (100%)	0.999
mTICI 3		57 (59.4%)	56 (60.9%)	52 (61.2%)	5 (45.5%)	4 (57.1%)	3 (100%)	0.630
ASITN/SIR (IQR)		2 (1, 3)	2 (0, 3)	2 (1, 3)	2 (1, 3)	1 (0,2)	0 (0, 1)	<0.001

mRS, modified Rankin Scale; sICH, symptomatic intracranial hemorrhage; TICI, Thrombolysis in Cerebral Infarction; ASITN/SIR, American Society of Interventional and Therapeutic Neuroradiology/Society of Interventional Radiology.

Of the 130 patients, 106 underwent EVT, among which 80% (85/106) had concordant indications for EVT in both profiles, with a 90-day functional independence rate of 55.3% after thrombectomy (47/85 patients; Table 2). The conditions of 18 (14%) patients were discordant, and the functional independence rate was 44% (8/18). 3% of the patients had no indication for EVT based on either method and did not achieve functional independence after EVT. On further analysis of these discordant profiles, six out of 11 (54.5%) patients with FVH-DWI mismatch and without perfusion mismatch achieved functional independence after EVT. In contrast, among patients with perfusion mismatch and without FVH-DWI mismatch, two out of seven (28.6%) achieved functional independence; however, the sample size for this inconsistency was very small.

## 4 Discussion

Our study showed that in the imaging evaluation of patients presenting in the 6–24 h time window, MRI-based triage (based on FVH-DWI mismatch) demonstrated moderate consistency in decision-making for EVT compared to perfusion-based triage. Moreover, no significant difference was observed in the 90-day functional independence rate and safety outcomes in EVT patients when screening based on either MRI or perfusion criteria. These results suggest that both MRI based on FVH-DWI mismatch and perfusion are efficient and equal in efficacy in preoperative imaging evaluation of endovascular thrombectomy in a 6–24 h time window.

According to the stringent image screening criteria of the DAWN and DEFUSE 3 trials, the thrombectomy time window was extended to 24 h (1, 2), shifting the perception from the traditional time window of acute stroke treatment to the concept of “mismatch” based on physiology to salvage penumbral brain tissue. Both the DAWN and DEFUSE 3 studies screened for “slow progression” individuals with small infarct cores and greater penumbra through the concept of “penumbra-core mismatch.” Researchers have noted that the stringent inclusion criteria of the DAWN and DEFUSE 3 trials constrained the selection of patients (14). During a single-center retrospective study, it was found that 70% of patients with acute ischemic stroke and anterior circulation large-vessel occlusion did not qualify for the inclusion criteria of the DAWN and DEFUSE 3 trials because of factors such as a large infarct core, a high mRS score, and no significant mismatch. Among them, 1/4 patients achieved a 30% functional independence rate after EVT (15). An additional multicenter retrospective study involved 21 patients who met the DAWN study’s inclusion criteria but underwent thrombectomy more than 24 h after onset. Of these, 81% achieved reperfusion and had a functional independence rate of 43% (16). In a multicenter multinational study, patients who were selected for EVT in the late time window based on non-contrast CT (NCCT) had outcomes similar to those of patients who were selected using CTP or MRI after adjusted analyses (17, 18). In the present study, FVH-DWI mismatch and perfusion screening showed moderate consistency (κ = 0.42, 95% CI, 0.17–0.67) in the preoperative evaluation for thrombectomy in patients with ischemic stroke and anterior circulation occlusion. Of the 130 patients, 87.7% (114/130) had an FVH-DWI mismatch, similar to a previous study (19). Despite this high level of concordance, 14% (18/130) of patients had discordant MRI and perfusion profiles. These results suggest that the image-screening method alone could lead to the exclusion of patients who may benefit from EVT.

Although the precise pathophysiological mechanism of FVH syndrome remains unclear, it can be divided into distal FVH syndrome (i.e., M3 and/or distal MCA, representing slow retrograde collateral circulation) and proximal FVH syndrome (i.e., stable and slow anterograde blood flow in the M1 and/or M2 regions) based on its location. Among these, distal FVH syndrome has more important clinical significance (20, 21). The correlations between FVH syndrome and acute arterial occlusion, chronic arterial stenosis, and ischemic collateral circulation are widely accepted. The FVH sign has a good diagnostic efficacy in identifying large-vessel occlusions, especially in MCA and ICA occlusions. An FVH exceeding the boundary of cortical injury in the DWI territory is considered to indicate an FVH-DWI mismatch. Recent studies have confirmed the predictive value of FVH-DWI mismatches in demonstrating a good clinical prognosis (22, 23). FVH-DWI mismatch can be used to quickly identify patients with anterior circulation large-vessel occlusion, good collateral circulation, and potential benefits from recanalization, and may become a reliable alternative to PWI-DWI mismatch (19). Our study showed that FVH-DWI mismatch had functional outcomes comparable with those of MRI and perfusion screening in the late time window (55.2% vs. 53.2%; *p* = 0.789).

In a further analysis of 18 discordant cases, six out of 11 (54.5%) patients with FVH-DWI mismatch and without perfusion mismatch achieved functional independence after EVT, with a mortality rate of 0. Surprisingly, only two out of seven (28.6%) patients had functional independence among those with perfusion mismatch and without FVH-DWI mismatch, with a mortality rate of 28.6%. In this group of patients, MRI demonstrated advantages over perfusion in terms of effectiveness and safety. In a further in-depth study of seven patients with perfusion mismatch but without FVH-DWI mismatch, the patients had a larger ischemic penumbra and a poorer NIHSS score improvement during hospitalization than those with FVH-DWI mismatch and no perfusion mismatch. More importantly, these patients had lower American Society of Interventional and Therapeutic Neuroradiology/Society of Interventional Radiology (ASITN/SIR) scores, indicating poorer collateral circulation. Numerous studies have verified that good collaterals are associated with a smaller core infarct upon presentation, slower infarct progression, and increased chances of achieving functional independence after 90 days in patients with acute ischemic stroke treated with EVT in the late time window (8). In addition, the perfusion examination cannot dynamically reflect the changes in the brain during acute ischemic stroke (24). At the same time, factors such as poor cardiac output, atrial fibrillation, severe proximal arterial stenosis or poor placement of arterial and venous density regions of interest can all affect perfusion results (25). Misjudgment of the core-to-penumbra ratio may result in misclassification of patients. In contrast, the FVH-DWI mismatch does not pose these limitations, in addition it integrates information about collateral status, which is a crucial determinant of infarct progression and functional outcome. Therefore, FVH-DWI may provide more accurate results in predicting functional outcomes and potentially reduce cases of futile recanalization compared to perfusion imaging alone. Recent studies have confirmed the advantages of magnetic resonance imaging (MRI) over perfusion-based imaging. A study on image-screening methods for the proportion of futile recanalization showed that MRI was linked to a lower risk of futile recanalization compared to CTP (26). Research on the Highly Effective Reperfusion evaluated in Multiple Endovascular Stroke Trials (HERMES) cohort showed that CTP was associated with a lower functional independence rate than MRI (27).

Previous trials have largely excluded patients with large infarct volumes on CTP or low CT ASPECTS (1, 2). In the present study, although two patients with infarct cores exceeding 70 mL did not meet the perfusion mismatch criteria, there was an FVH-DWI mismatch. All patients achieved functional independence after EVT without sICH or neurological worsening. In a recent HERMES meta-analysis, patients with ASPECTS scores of 3–5 and an infarct core greater than 70 mL showed improved prognosis with EVT, but the risk of symptomatic ICH increased (27). In the ANGEL-ASPECT trial, endovascular treatment was superior to medical management alone; however, more cases of intracranial hemorrhage (28). Therefore, FVH-DWI mismatch may be a more inclusive criterion, allowing for intervention in more patients.

As a retrospective analysis, this study has some limitations, including susceptibility to selection bias. Furthermore, our sample size was relatively small, with certain subgroups, particularly those with unfavorable MRI and perfusion profiles, having smaller sample sizes. Therefore, the estimation of the event occurrence rate may have been inaccurate. As such, accurate conclusions could not be drawn for these subgroups. MRI may not be readily available at many centers for stroke triage. Hence, the use of NCCT may be of interest for triage in centers with limited resources (4). Considering the large positive ischemic core data for EVT (29), it is not known how triage based on FVH-DWI mismatch will be compared in the selection of patients with large ischemic core infarctions. Prospective, multicenter, large-sample, homogeneous, randomized controlled trials will be needed to confirm the results of this study.

## 5 Conclusion

This study demonstrated that in the imaging evaluation of patients who underwent thrombectomy in the 6–24 h time window, MRI-based triage using FVH-DWI mismatch showed moderate consistency in decision-making for EVT compared with perfusion-based triage. In fact, no significant difference was observed in the 90-day functional independence rate and safety endpoints between selection based on MRI and perfusion after EVT. Therefore, MRI imaging based triaging by FVH-DWI mismatches may be an effective and reliable approach for selecting patients for endovascular thrombectomy in a late time window.

## Data availability statement

The original contributions presented in the study are included in the article/supplementary material, further inquiries can be directed to the corresponding author.

## Ethics statement

The studies involving humans were approved by the Ethics Committee of Tianjin Huanhu Hospital. The studies were conducted in accordance with the local legislation and institutional requirements. The participants provided their written informed consent to participate in this study. Written informed consent was obtained from the individual(s) for the publication of any potentially identifiable images or data included in this article.

## Author contributions

LL: Writing – review & editing, Writing – original draft, Methodology, Data curation. GZ: Writing – original draft, Software, Formal analysis, Data curation. FM: Writing – review & editing, Resources, Methodology, Formal analysis. SL: Writing – original draft, Resources, Methodology, Formal analysis. SW: Writing – original draft, Software, Methodology, Investigation. YD: Writing – original draft, Resources, Investigation. DL: Writing – original draft, Visualization, Supervision, Project administration. MW: Writing – review & editing, Supervision, Resources, Methodology, Investigation, Data curation, Conceptualization.
